# Hepatic Overexpression of GRP94 in a Rabbit Model of Parenteral Nutrition-Associated Liver Disease

**DOI:** 10.1155/2015/269831

**Published:** 2015-03-30

**Authors:** Xueping Zhu, Xiaomin Zhang, Lingling Yu, Yumin Xu, Xing Feng, Jian Wang

**Affiliations:** ^1^Department of Neonatology, Children's Hospital Affiliated to Soochow University, Suzhou, Jiangsu 215003, China; ^2^Department of Neonatology Surgery, Children's Hospital Affiliated to Soochow University, Suzhou, Jiangsu 215003, China

## Abstract

*Objective*. To use a rabbit model of parenteral nutrition-associated liver disease (PNALD) to study changes of the endoplasmic reticulum stress (ERS) marker glucose regulatory protein 94 (GRP94) and determine its role in the pathogenesis of PNALD. *Methods*. A rabbit PNALD model total parenteral nutrition (TPN) group was established. A corresponding control group received breast-feeding for one week. Serum biochemical parameters were measured and liver histological examinations were performed. The level of GRP94 mRNA and protein were measured. *Results*. The results showed that the serum TBIL, DBIL, and *γ*-GT levels in the TPN group were significantly higher than those in the control group, while levels of serum ALB in TPN group were significantly lower than those in the control group. The immunohistochemistry results showed that the protein expression level of GRP94 in the liver of TPN group was significantly increased compared with the control group. The RT-PCR results showed that the level of GRP94 mRNA in the liver of the TPN group was significantly higher compared with the control group. *Conclusions*. The mRNA and protein levels of GRP94 in the TPN group were both significantly increased, indicating that ERS may be directly related to the occurrence and development of PNALD.

## 1. Introduction

Parenteral nutrition has brought about a revolutionary improvement in the care of neonates with growth failure due to intestinal dysfunction. Since the first reported case of long-term parenteral nutrition supplement in a newborn girl in the United States in the 1960s, there have been more than 30,000 patients whose survival has depended on parenteral nutrition [1]. However, long-term (>2 weeks) parenteral nutrition is associated with a number of problems. Parenteral nutrition-related liver disease (PNALD) is one of the most serious complications of neonatal parenteral nutrition-associated liver disease, which usually presents with steatosis, cholestasis, and elevated aminotransferases. It has been reported [2] that about 30%–60% of children on long-term parenteral nutrition develop PNALD. Children born prematurely are more likely to suffer from total parenteral nutrition-associated cholestasis (PNAC) [3, 4] and even severe life-threatening cirrhosis. Despite the seriousness of the disease, the specific etiology and pathogenesis remains unclear.

Because hepatocytes have highly active protein synthetic activity and abundant endoplasmic reticulum, it is possible that endoplasmic reticulum stress response (ERS) may be involved. ERS has been shown to be involved in pathological changes of various liver diseases [5, 6]. ERS has been implicated in the development of nonalcoholic steatohepatitis [7], alcoholic liver disease [8], ischemia/reperfusion liver injury [9], and cholestatic disease [10]. We hypothesized that ERS could also play an important role in development of PNALD. Glucose-regulated protein 94 (GRP94) is an important marker protein of ERS and a chaperone localized in the endoplasmic reticulum [11, 12]. As one of the known endoplasmic reticulum stress proteins, GRP94 has been shown to be important contributor to correct protein folding and processing, maintaining the stability of the endoplasmic reticulum under stress and generally protecting cells [12–14]. Thus, GRP94 could well be an indicator of ERS in liver disease. The aim of this study was to use a rabbit PNALD model to study the changes in expression the ERS marker protein GRP94 and determine its role in the pathogenesis of PNALD.

## 2. Materials and Methods

### 2.1. The PNALD Model and Experimental Groups

Sixteen 7-day-old breastfed New Zealand White rabbits, obtained from Wuxi Huishan Jiangnan Experimental Animal Center, were randomly divided into a total parenteral nutrition group (TPN group, *n* = 8) and a control (breastfed) group (*n* = 8). The PNALD rabbit model was established as described previously [15, 16] with some modifications. Briefly, the TPN group received continuous total parenteral nutrition 240 mL/kg·d for each rabbit, consisting of formula composition shown in Table 1. Formula, 240 mL, was introduced through a silastic catheter inserted in the right jugular vein. TPN group received 40 mL of a 20% medium- and long-chain fat emulsion (72 kcal, 34.3% of total calories), consisting of 2 g soy bean oil, 2 g medium chain triglyceride, and 0.24 g egg phospholipids; 80 mL of 11.4% pediatric compound amino acid injection-18AA-II (36.4 kcal, 17.3% of calories); 36 mL of 50% glucose (72 kcal, 48.4% of calories); 74 mL of 10% glucose (29.6 kcal); 4 mL of 10% NaCl; 3 mL of 10% KCl; 3 mL of 10% calcium gluconate; half of a water soluble and fat-soluble vitamin ampoule originally containing 0.3 mg vitamin B1, 0.36 mg vitamin B2, 4 mg nicotinamide, 0.4 mg vitamin B6, 1.5 mg pantothenic acid, 10 mg vitamin C, 6 *μ*g biotin, 40 *μ*g folic acid, 0.5 *μ*g vitamin B12, 25 *μ*g (82.5 IU) vitamin A, 10.125 *μ*g (5 IU) vitamin D, 0.2275 mg (0.25 IU) vitamin E, and 3.75 *μ*g vitamin K1; and trace elements CaCl_2_·2H_2_O, 39.25 mg; MgCl_2_·6H_2_O, 15.21 mg; FeCl_3_·6H_2_O, 0.675 mg; ZnCl_2_, 0.135 mg; MnCl_2_·4H_2_O, 0.395 mg; CuCl_2_·2H_2_O, 42.5 *μ*g; NaF, 0.105 mg; and KI, 8.5 *μ*g. Each 240 mL portion of TPN comprised 210 kcal and the ratio of sugar to lipid was 1.4 : 1. The components in the mixture were purchased from Sino-Swed Pharmaceutical, China.

The control group received breast-feeding. Both groups were treated for one week and housed under conditions of constant temperature of 26–28°C, relative humidity of 40–60%, and 12 h light and 12 h dark. The study protocol was approved by the Animal Care Committee of the Children's Hospital Affiliated to Soochow University.

### 2.2. Specimen Collection and Processing

All animals were anesthetized with 10% chloral hydrate by intraperitoneal injection and fixed on the dissecting table. The precordium was shaved and disinfected with iodine alcohol. The point of maximal impulse was identified and 2 mL blood was obtained by cardiac puncture and transferred to the anticoagulant tubes, centrifuged at 3500 rpm serum, and stored at −20°C freezer. The animals were killed by an overdose of anesthetic and the abdominal cavity was quickly opened. Liver tissue was excised and cleaned with normal saline. Some tissue was fixed in 10% paraformaldehyde and 50–100 mg tissue samples were placed in tube and stored in liquid nitrogen until analyzed.

### 2.3. Blood Biochemical Tests

Serum total bilirubin (TBIL, *μ*M), bilirubin (DBIL, *μ*M), alanine aminotransferase (ALT, IU/L), aspartate aminotransferase (AST, IU/L), total protein (TP, g/L), albumin (ALB, g/L), *γ*-glutamyl peptidase (*γ*-GT, IU/L), alkaline phosphatase (ALP, IU/L), triglyceride (TG, mM), total cholesterol (TC, mM), and prealbumin (PA, mg/L) were measured by a Hitachi 7600 automatic biochemical analyzer (Japan).

### 2.4. Liver Pathology

Fresh liver tissue was fixed with 10% paraformaldehyde and dehydrated with serial different concentrations of alcohol. The dehydrated liver tissue was clear through xylene and embedded by paraffin. The paraffin-embedded tissue blocks were cut into 5-micron thick slices, which were applied onto glass slides and dried in 45°C incubator. The glass slides were dewaxed and stained with hematoxylin and eosin (H and E).

### 2.5. Measurement of GRP94 mRNA Levels in Liver Tissue by RT-PCR

Frozen liver tissue was thawed at room temperature and ground in DEPC-treated mortar. Total RNA was obtained using a Trizol extraction kit. RT-PCR was done using a Promega reverse transcription kit. Primers were designed using primer 5.0 software and synthesized by Shanghai Sangon Biological Engineering Company after a GenBank Blast search for homology. All operations were carried out according to the kit instructions. Rabbit GAPDH was selected as internal reference whose expected fragment size was 497 bp. The primers were forward: 5′-GTTTGTGATGGGCGTGAA-3′; reverse: 5′-CGAAGGTAGAGGAGTGGGTG-3′. GRP94 fragment size was 583 bp and primers were forward: 5′-AGGAAACACTCTGGGACG-3′; reverse: 5′-ATTCAGGTACTTAGGCATC-3′. RT-PCR products were observed on a 1.5% agarose gel electrophoresis. Semiquantitative analysis was made in Bio2239 gel imager (Bio-Print Company).

### 2.6. GRP94 Protein Levels in Liver Tissue as Determined by Immunohistochemistry

Immunohistochemical analyses were conducted using a streptomyces avidin-peroxidase link method. All slides were pretreated with polylysine (Boster Biological Engineering Co., Ltd.). Cells with brownish yellow granules in the cytoplasm were considered to be positive. Liver tissue slices in glass slides were observed by light microscopy after immunohistochemical staining according to the manufacturer's instructions (Suzhou En Maike Biotechnology Co., Ltd.). Three nonoverlapping fields of view at high magnification (×400) were randomly selected from each slide for gray degree scanning using Image-Pro-Plus image analysis software system. The average gray level of each group was calculated to reflect the positive intensity of GRP94.

### 2.7. Statistical Analysis

Statistical analysis was made using SPSS17.0 statistical software. Quantitative data were described as mean ± standard deviation. Comparisons between two groups of quantitative variables were performed using Student's *t*-test. *P* values < 0.05 were considered to indicate statistical significance.

## 3. Results

### 3.1. Biochemical Parameters in the TPN and Control Groups

**Figure 1:**
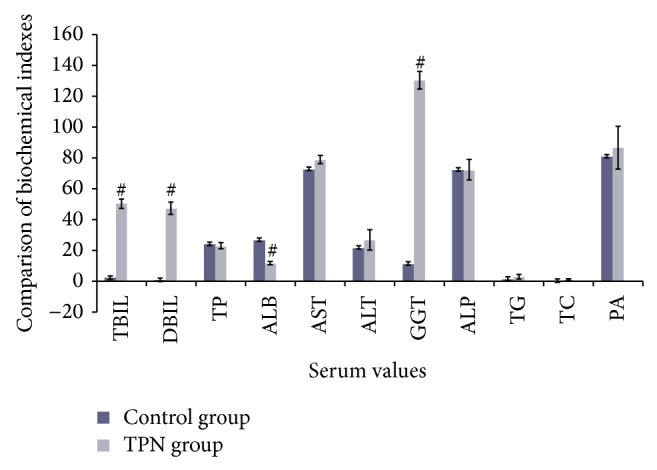
A comparison of serum biochemical data from the TPN and control groups. Note: each value is the mean ± SD of assays using 8 independent samples. Compared with the control group, ^#^ *P* < 0.01. TBIL: total bilirubin, DBIL: direct bilirubin, TP: total protein, ALB: albumin, AST: aspartate aminotransferase, ALT: alanine aminotransferase, r-GT: r-glutamyl GGT, ALP: alkaline phosphatase, TG: triglycerides, TC: total cholesterol, and PA: prealbumin.

There were statistically significant differences in serum levels of TBIL (*t* = 41.59, *P* < 0.01), DBIL (*t* = 33.38, *P* < 0.01), *γ*-GT (*t* = 39.07, *P* < 0.01), and ALB (*t* = −12.36, *P* < 0.01) between the two groups. However, there were no statistically significant differences in serum TP, ALT, AST, ALP, TG, TC, or PA (*P* > 0.05). Compared to the control group, serum TBIL, DBIL, and *γ*-GT levels in the TPN group were significantly higher (*P* < 0.01), while ALB was significantly lower (*P* < 0.01). These results are shown in Table 2 and Figure 1.

### 3.2. Liver Histology in the TPN and Control Groups

**Figure 2:**
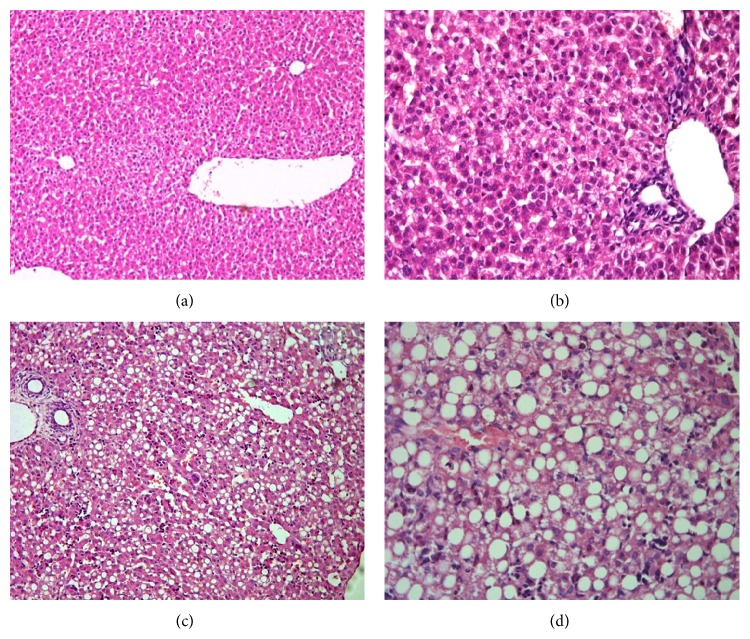
Representative sections of livers from the TPN and control groups obtained on d 7 and stained with H and E. Note: (a) control group, 200x; (b) control group, 400x; (c) TPN group, 200x; and (d) TPN group, 400x.

The liver tissue of the control group showed morphological normal hepatocytes, with no bile duct abnormalities, inflammatory cell infiltration, or hepatocyte degeneration and necrosis, as shown in Figures 2(a) and 2(b). In contrast, in liver tissue of the TPN, there were inflammatory cell infiltration, diffuse steatosis, and liver cell cord structural disorder. However, there were no bile duct dilatation or epithelial hyperplasia, no significant cholestasis, and no visible fibrosis group with lobular structure, as shown in Figures 2(c) and 2(d).

### 3.3. Levels of Liver GRP94 Protein in the TPN and Control Groups

**Figure 3:**
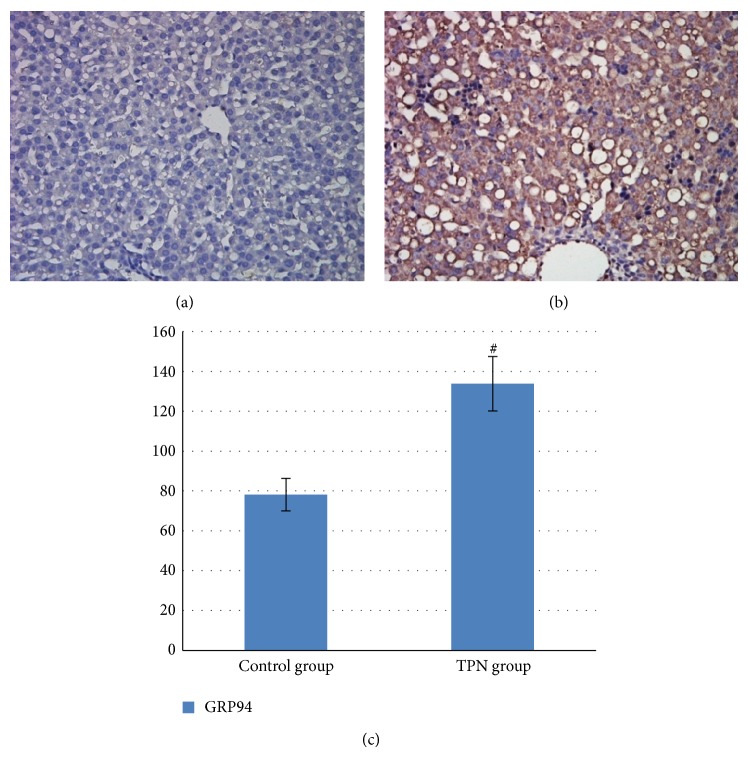
Representative immunohistochemical staining of GRP94 protein in liver tissue from the TPN and control groups. Note: (a) control group, 400x; (b) TPN group, 400x; and (c) a GRP94 protein expression gray value histogram of liver tissue from the two groups. Compared with the control group, ^#^ *P* < 0.01.

Immunohistochemistry showed that GRP94 protein expression gray values in the TPN group and the control group were 133.838 ± 13.66, 78.138 ± 8.169, respectively. GRP94 protein levels in the TPN group were significantly higher than those in the controls (*P* < 0.01) as shown in Figures 3(a), 3(b), and 3(c) and Table 3.

### 3.4. Liver GRP94 mRNA Levels in the TPN and Control Groups

**Figure 4:**
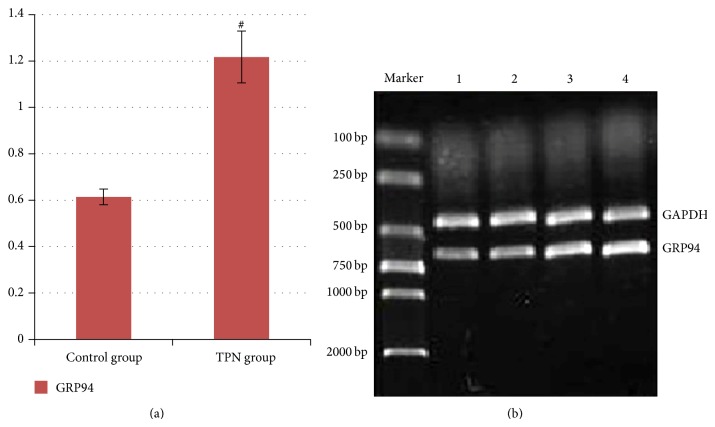
(a) A gray value histogram of GRP94 mRNA levels in liver tissue from the two groups. Compared with the control group, ^#^ *P* < 0.01. The bands were quantified as the relative integrated optical density (IOD) values of the ratio of GRP94/GAPDH for two groups, mean ± SD, *n* = 8. (b) Liver tissue GRP94 mRNA amplified by RT-PCR and analyzed by electrophoresis. Lanes: marker, DNA marker; 1 and 2, control group; 3 and 4, TPN group.

RT-PCR in the liver tissue showed that the GRP94 mRNA expression gray values in the TPN group and the control group were 1.217 ± 0.112 and 0.614 ± 0.034, respectively. GRP94 mRNA expression gray values in the TPN group were significantly higher than those of controls (*P* < 0.01) as shown in Table 4 and Figure 4.

## 4. Discussion

Parenteral nutrition has offered powerful nutritional support for critically ill infants including those who fail to get enteral nutrition. But at the same time, there are also negative effects during long-term parenteral nutrition, which are mainly PNALD occurrence [17]. However, the etiology and pathogenesis of PNALD are poorly understood [18–21]. Hepatocytes perform a myriad of metabolic functions and thus are enriched in both smooth and rough ER. Recently, ERS response has been observed in a variety of liver diseases and ERS response accompanies nearly all forms of acute and chronic liver disease [5–10]. Some of these observations offer mechanistic insights and present potential therapeutic targets. However, it is not known whether ERS response also plays an important role in PNALD. These associations of ERS response and other liver diseases also may indicate that a new hypothesis is required to test the role of the ERS in PNALD. To test the hypothesis, the expression changes of GRP94, which is one of ERS marker proteins, were analyzed in a rabbit PNALD model.

ERS has been shown to be involved in preventing protein misfolding and unfolding, thus contributing to the maintenance of cell survival and normal function [22]. However, ERS of long duration can induce apoptosis [22]. Correct protein folding within the endoplasmic reticulum requires the assistance of chaperone proteins, such as Bip/GRP78 and GRP94, and folding enzymes [23]. Under normal conditions, the endoplasmic reticulum chaperones Bip/GRP78, GRP94 and Ire1, ATF6, and PERK combine to form a stable complex and stays in the endoplasmic reticulum lumen. When protein unfolding occurs, a large number of these unfolded or misfolded proteins accumulate resulting in dissociation of Ire1, ATF6, PERK, and BIP [23]. Excessively long or strong ERS can increase the levels of ERS-related protein GRP94 [24]. It is widely accepted that GRP94 is an ERS marker [25]. Therefore, GRP94 was selected for detecting the occurrence of ERS in PNALD.

In the current study, after one week of intravenous nutrition in 7-day-old rabbits, serum TBIL, DBIL, and *γ*-GT in the TPN group were significantly higher (*P* < 0.01), while ALB was significantly lower (*P* < 0.01) than that in control group. Liver pathology showed inflammatory cell infiltration, diffuse steatosis, and liver cell cord structural disorder in the TPN group, while these changes were not observed in the control group. Liver damage occurred after 1 week of TPN that was consistent with previous research results [16, 17]. The immunohistochemistry results showed that the protein expression level of GRP94 in the liver of TPN group was significantly increased compared with the control group (133.838 ± 13.664 versus 78.138 ± 8.169) (*P* < 0.01). The RT-PCR results showed that the expression level of GRP94 mRNA in the liver of the TPN group was significantly increased compared with the control group (1.217 ± 0.113 versus 0.614 ± 0.034) (*P* < 0.01). Therefore, the mRNA and protein expression of GRP94 in the TPN group were both significantly increased, which indicated that ERS may be directly related to the occurrence and development of PNALD.

In the PNALD model, *γ*-GT activity, which is mainly attributed to the hepatobiliary system [26], has been reported to be significantly increased. While TBIL was also significantly increased, the largest contribution to the elevated TBIL was DBIL but not IBIL. Hyperbilirubinemia with mainly DBIL elevation often suggests bile duct injury or obstruction. An elevation of GGT which has been reported to be closely related to hepatic steatosis [27–29] was also observed in liver pathology results of the current study. With severe liver damage, synthesis, intracellular transport, and release of ALB can be affected resulting in decreased serum ALB [30]. Therefore, long-term parenteral nutrition may cause inflammatory cell infiltration, diffuse steatosis, and liver cell cord structural disorder in liver. This liver pathology change may be accompanied with elevated serum DBIL and *γ*-GT and decreased serum ALB. Therefore, we speculate that increases in TBIL, DBIL, and *γ*-GT and the reduction of ALB levels may be early indicators of the development of PNALD.

There are limitations to this study. The number of experiments was small, and the experimental TPN application period was not long enough to observe cholestatic changes. Future experiments should be done for longer durations of treatment to observe the relations of relevant biochemical and the occurrence and development of PNALD. There is, however, a problem related with the specificity of these results for parenteral nutrition therapy since controls for the stress were not included (separation from the mother, anesthesia, catheter inserted in the right jugular vein, infusion, etc.). This limitation is likely not significant based on our previous report that soybean oil parenteral nutrition was associated with significant liver dysfunction, as indicated by higher serum total bilirubin, direct bilirubin, and *γ*-GT and lower serum albumin levels compared to control. These effects were not observed in the fish oil fat emulsion group (TPN-FO), which was similar to the control. Moreover, histological examination of liver tissues revealed hepatic damage in the soybean fat emulsion group (TPN-soy) not seen in the TPN-FO, including inflammatory cell infiltration, diffuse hepatic steatosis, and disrupted hepatic cord structure [31].

## 5. Conclusion

In conclusion, the current study showed that the mRNA and protein levels of GRP94 in the TPN group were both significantly increased compared with those in the control group. The differences were statistically significant (*P* < 0.05). These results indicate that ERS occurred in the TPN group and may be involved in the development of PNALD. This information may provide an important novel basis for the detection and prevention of PNALD.

## Figures and Tables

**Table 1:** Total parenteral nutrition solution formula (total liquid volume 240 mL·kg^−1^·d^−1^ and total calories 210 kcal·kg^−1^·d^−1^).

Ingredient	Volume (mL)	Calories (kcal)	Calories percentage (%)
20% medium/long-chain fat emulsion^(1)^	40.0	72.0	34.3
11.4% compound amino acids	80.0	36.4	17.3
50% glucose	36.0	72.0	48.4
10% glucose	74.0	29.6	
10% sodium chloride	4.0		
10% potassium chloride	3.0		
10% calcium gluconate	3.0		
Water-soluble vitamins^(2)^	1/2 ampoule		
Fat-soluble vitamins^(3)^	1/2 ampoule		

Total	240.0	210.0	

Note: ^(1)^medium/long-chain fat emulsion (250 mL) composition: soybean oil 12.5 g, medium chain triglycerides 12.5 g, and lecithin 1.5 g. ^(2)^Water-soluble vitamins composition: vitamin B 10.6 mg, vitamin B 20.72 mg, nicotinamide 8 mg, vitamin B 60.8 mg, pantothenic acid 3 mg, vitamin C 20 mg, biotin 12 *μ*g, folic acid 80 *μ*g, and vitamin B 121 *μ*g. ^(3)^Fat-soluble vitamins composition: vitamin A 50 *μ*g (165 IU), vitamin D 20.25 *μ*g (10 IU), vitamin E 0.455 mg (0.5 IU), and vitamin K1 7.5 *μ*g.

**Table 2:** Comparison of biochemical indicators between the groups.

Grouping	TBIL	DBIL	TP	ALB	AST	ALT	r-GT	ALP	TG	TC	PA
	(umol/L)	(umol/L)	(g/L)	(g/L)	(IU/L)	(IU/L)	(IU/L)	(IU/L)	(mmol/L)	(mmol/L)	(mg/L)
Control group	2.28 ± 1.04	1.05 ± 0.57	24.25 ± 3.12	26.86 ± 2.83	72.75 ± 10.33	21.93 ± 1.49	11.28 ± 5.68	72.39 ± 13.68	1.74 ± 1.29	0.41 ± 0.21	80.86 ± 29.99
TPN group	50.25 ± 3.06^#^	47.28 ± 3.91^#^	23.02 ± 1.94	11.73 ± 1.05^#^	78.85 ± 2.70	26.83 ± 6.57	130.47 ± 5.61^#^	72.30 ± 6.69	2.86 ± 1.49	0.92 ± 0.62	86.51 ± 13.85
*t* value	41.59	33.38	−0.85	−12.36	1.39	2.07	39.07	0.01	−0.44	−0.74	−0.83
*P* value	<0.01	<0.01	0.41	<0.01	0.19	0.06	<0.01	0.98	0.67	0.48	0.42

Note: (1) compared with the control group, ^#^ *P* < 0.01.

(2) TBIL: total bilirubin, DBIL: direct bilirubin, TP: total protein, ALB: albumin, AST: aspartate aminotransferase, ALT: alanine aminotransferase, r-GT: r-glutamyl GGT, ALP: alkaline phosphatase, TG: triglycerides, TC: total cholesterol, and PA: prealbumin.

**Table 3:** GRP94 protein expression gray values in liver tissues (±*s*).

Groups	GRP94 protein expression gray values ($\overset{-}{x}\pm s$)
Control group (*n* = 8)	78.138 ± 8.169
TPN group (*n* = 6)	133.838 ± 13.664^#^
*t* value	−9.546
*P* value	<0.01

Note: compared with the control group, ^#^ *P* < 0.01.

**Table 4:** Comparison of liver tissue GRP94 mRNA level gray values ($\overset{-}{x}\pm s$).

Groups	GRP94 mRNA
Control group (*n* = 8)	0.614 ± 0.034
TPN group (*n* = 6)	1.217 ± 0.112^#^
*t* value	−14.427
*P* value	<0.01

Note: compared with the control group, ^#^ *P* < 0.01.
